# Glucocorticoids, antenatal corticosteroid therapy and fetal heart maturation

**DOI:** 10.1530/JME-18-0077

**Published:** 2018-05-02

**Authors:** Emma J Agnew, Jessica R Ivy, Sarah J Stock, Karen E Chapman

**Affiliations:** 1 University/BHF Centre for Cardiovascular ScienceUniversity of Edinburgh, The Queen’s Medical Research Institute, Edinburgh, UK; 2 MRC Centre for Reproductive HealthUniversity of Edinburgh, The Queen’s Medical Research Institute, Edinburgh, UK

**Keywords:** antenatal corticosteroid, glucocorticoid, steroid, preterm birth, 11β-HSD, fetal origins, programming, HPA axis, lung, heart

## Abstract

Glucocorticoids are essential in mammals to mature fetal organs and tissues in order to survive after birth. Hence, antenatal glucocorticoid treatment (termed antenatal corticosteroid therapy) can be life-saving in preterm babies and is commonly used in women at risk of preterm birth. While the effects of glucocorticoids on lung maturation have been well described, the effects on the fetal heart remain less clear. Experiments in mice have shown that endogenous glucocorticoid action is required to mature the fetal heart. However, whether the potent synthetic glucocorticoids used in antenatal corticosteroid therapy have similar maturational effects on the fetal heart is less clear. Moreover, antenatal corticosteroid therapy may increase the risk of cardiovascular disease in adulthood. Here, we present a narrative review of the evidence relating to the effects of antenatal glucocorticoid action on the fetal heart and discuss the implications for antenatal corticosteroid therapy.

## Introduction

Through most of gestation, the mammalian fetus is maintained in a low glucocorticoid environment, with fetal glucocorticoid concentrations typically five- to ten-fold lower than maternal. As birth approaches, fetal plasma glucocorticoid concentration rises dramatically. This is essential for survival once born. Without glucocorticoid action, the lungs, heart and other organs and tissues are immature at birth, resulting in neonatal death (Fowden *et al*. 1998). Preterm birth (prior to 37 weeks completed gestation) occurs before this physiological rise in endogenous glucocorticoids. Hence, antenatal corticosteroid therapy (ACT) – in which a potent synthetic glucocorticoid, betamethasone or dexamethasone, is administered to women at risk of preterm delivery – is widely used to mature the fetal lungs. This aims to reduce neonatal morbidity and improve survival of preterm babies. However, whether ACT faithfully mimics the effects of endogenous glucocorticoids on the maturation of other fetal organs, including the heart, is currently unclear. Crucially, less than half of ACT administered prior to preterm birth is optimally timed, and around 50% of women who receive ACT go on to deliver at or near term (Razaz *et al*. 2015, Makhija *et al*. 2016, Grzeskowiak *et al*. 2017) (reviewed in Kemp *et al*. 2016). Given evidence associating excessive prenatal glucocorticoid exposure with long-term adverse cardiovascular consequences (Fowden *et al*. 2016), it is important to establish exactly how fetal glucocorticoids – either endogenous or exogenous – ‘programme’ cardiovascular health in later life. This knowledge is essential to allow an informed analysis of the benefits and harms resulting from ACT and to refine the use of ACT during pregnancy.

## Endogenous glucocorticoids and fetal maturation

The adrenal gland is the site of synthesis and release of corticosteroid hormones: mineralocorticoids and the glucocorticoids, cortisol and corticosterone (cortisol predominates in most mammals, whereas rats and mice produce only corticosterone). In human embryos, the adrenal cortex is discernible from the eighth week of gestation, though the morphology differs from the adult (Mesiano & Jaffe 1997). Prior to birth, the fetal zone (comprising most of the adrenal cortex) is the major site of steroidogenesis. The definitive zone starts to produce mineralocorticoids in late gestation, whereas the transitional zone produces cortisol, transitioning into the zona fasciculata, the site of glucocorticoid production, from late gestation (Mesiano & Jaffe 1997). *De novo* synthesis of cortisol from cholesterol initiates in the human fetal adrenal gland around the 28th week of gestation (Mastorakos & Ilias 2003), but plasma glucocorticoid concentrations remain low until the week before birth (Fowden *et al*. 1998). Similarly, in fetal mice, plasma glucocorticoid concentrations are low during early-to-mid-gestation then increase rapidly following initiation of adrenal steroidogenesis at embryonic day (E) 14.5 (Michelsohn & Anderson 1992), approximately 5 days before birth. The low glucocorticoid environment during gestation is maintained by at least two placental mechanisms: 11β-hydroxysteroid dehydrogenase (11β-HSD)-2, which converts active cortisol and corticosterone into their intrinsically inert 11-keto-metabolites (Chapman *et al*. 2013) and P-glycoprotein-mediated active retrograde transport of glucocorticoids from fetus to mother, maintaining the feto–maternal glucocorticoid gradient in both endogenous and exogenous glucocorticoids (Varma 1986, Fowden & Forhead 2004, Walker *et al*. 2017). 11β-HSD2 is also widely expressed in the fetus during early-to-mid-gestation, to ‘mop-up’ any glucocorticoid that reaches sensitive tissues, particularly the brain (Wyrwoll *et al*. 2011, 2015). In late gestation, placental 11β-HSD-2 activity markedly declines (Brown *et al*. 1996, Murphy & Clifton 2003) and maternal glucocorticoid concentrations rise, coincident with increased fetal production of glucocorticoids (Mastorakos & Ilias 2003). Together, these generate the dramatic rise in fetal plasma glucocorticoid concentrations close to term. These increased concentrations of endogenous glucocorticoids act, via glucocorticoid receptor (GR), to mature the fetal lungs (Jobe & Ikegami 2000, Bird *et al*. 2015) and other organs, in order to survive in the extra-uterine environment after birth. Mice with global knockout of GR die shortly after birth with immature and severely impaired lung function (Cole *et al*. 1995). A proportion of GR knockout mice die earlier, in late gestation, with immature and poorly functional hearts (Rog-Zielinska *et al*. 2013), suggesting cardiac immaturity contributes to perinatal mortality and morbidity with deficiency in glucocorticoid action.

## ACT: a life-saving therapy

In high-income countries, and increasingly in low- and middle-income countries, antenatal corticosteroids (24 mg betamethasone or dexamethasone, administered over 48 h) are routinely administered to women considered at imminent risk of preterm delivery (birth prior to 37 weeks gestation and before the natural increase in endogenous glucocorticoid concentrations would be expected). Antenatal corticosteroids are widely accepted to be the most effective therapy to reduce neonatal morbidity and mortality in preterm infants born between 24 and 34 weeks gestation: ACT reduces the incidence of neonatal death and the incidence and severity of respiratory distress syndrome, cerebral haemorrhage and necrotising enterocolitis (Roberts *et al*. 2017). Thus, in high-income settings, antenatal corticosteroids are undoubtedly life-saving in preterm infants delivered at 24–34 weeks of gestation within a 2- to 7-day window following initiation of a single course of ACT. However, the safety and efficacy of ACT in other settings have been called into question: most notably in low- and middle-income settings, following multiple courses of ACT, in late preterm infants (born at 34–37 weeks of gestation) and where birth occurs outside the 2- to 7-day window following steroid administration (reviewed in Kemp *et al*. 2016). The latter is a particular concern in high-income settings where estimates suggest 50–80% of pregnant women that receive ACT remain undelivered at 7 days after treatment (McPheeters *et al*. 2005, Razaz *et al*. 2015), and 50% actually go on to deliver their babies at or near term (Razaz *et al*. 2015) (reviewed in Kemp *et al*. 2016). This suggests widespread over-treatment with antenatal corticosteroids resulting in unnecessary and inappropriately timed corticosteroid exposure in babies, many of which subsequently deliver at term. Similar concerns surround the use of repeat courses of ACT, administered when birth does not occur within 7 days of the first course and preterm delivery remains likely (McKinlay *et al*. 2015*b*, Kemp *et al*. 2016). There are potential short-term and long-term adverse effects associated with unnecessary or inappropriately timed corticosteroid treatment. Delivery later than 7 days after a single course of ACT is associated with an increased risk of perinatal death and maternal infection (McLaughlin *et al*. 2003). Some follow-up studies have demonstrated adverse neurodevelopmental effects in children born preterm or exposed to multiple courses of ACT (Asztalos *et al*. 2014, Crowther *et al*. 2016). Whether ACT affects risk of cardiovascular disease in humans remains unclear (Dalziel *et al*. 2005, de Vries *et al*. 2008, McKinlay *et al*. 2015*a*). However, cardiovascular disease can take decades to manifest, and it may yet be too early to fully assess the risk in adults. Nevertheless, excessive or mistimed exposure to glucocorticoids *in utero* is widely acknowledged to have detrimental long-term adverse effects in animals, including non-human primates, that increase the risk of cardiovascular and/or metabolic disease in adulthood (Seckl & Holmes 2007, Rog-Zielinska *et al*. 2014). The mechanisms remain to be fully elucidated, but undoubtedly include long-term adverse effects on the heart and vasculature.

## Maturation of the heart, before and after birth

In the time shortly before and continuing after birth, the heart undergoes extensive growth and remodelling, driven by cardiomyocyte hyperplasia and associated with structural, functional and biochemical maturation. These remarkable changes are driven by mechanical and hormonal factors and are crucial for survival following birth. They also set the foundation for later life, and, importantly, influence the risk of developing heart disease in adulthood. Increased cardiac load stimulates fetal myocyte proliferation (Sedmera *et al*. 2003, Drenckhahn 2009). The fetal heart needs to pump against the resistance of the placental vascular bed with the resulting haemodynamic forces being important drivers of cardiac maturation. The complexity of the fetal part of the placental vasculature increases through branching morphogenesis, though exactly how this influences fetal haemodynamics is currently unclear. In sheep, fetal hypertension promotes cardiomyocyte proliferation prior to terminal differentiation. Conversely, reduced systolic load reduces cardiomyocyte proliferation and, thus, fetal heart weight (reviewed in Thornburg *et al*. 2011). However, placental insufficiency, with high placental impedance against pulsatile flow, also impairs myocardial maturation (Thornburg *et al*. 2010); the mechanism remains to be fully elucidated.

Fetal systolic and diastolic functions improve before and shortly after birth, with increasing maturation of the contractile and relaxation properties of cardiomyocytes (Harada *et al*. 1997, Corrigan *et al*. 2010). It is proliferation of differentiated mononuclear diploid cardiomyocytes that increases heart mass in late gestation and early after birth in mice and sheep (Soonpaa *et al*. 1996, Porrello *et al*. 2011, Alkass *et al*. 2015, Jonker *et al*. 2015). The very limited evidence available is consistent with a similar capacity for cardiomyocyte proliferation in neonatal human hearts (Mollova *et al*. 2013). Beyond the first postnatal week in mice, cardiomyocyte proliferation is negligible, and increases in heart mass occur through cardiomyocyte hypertrophy following binucleation and terminal differentiation of cardiomyocytes. Any subsequent proliferation that occurs is restricted to rare mononucleated diploid cardiomyocytes (Patterson *et al*. 2017). Structural changes that occur in cardiomyocyte maturation during the neonatal period include increased myofibril density and organisation, maturation of mechanical and electrical coupling between cardiomyocytes and the appearance of binucleated cells. This latter is associated with a wave of DNA synthesis that occurs in mouse cardiomyocytes between postnatal day P4 and P7 (Soonpaa *et al*. 1996). Additionally, as perinatal cardiomyocytes mature, some nuclei become polyploid. In mice, increases in ploidy account for all cell-cycle activity following P13 (Brodsky *et al*. 1980, Alkass *et al*. 2015). The majority of murine cardiomyocyte nuclei are diploid, with only 10% being polyploid (Adler *et al*. 1996). Similarly, in humans, most cardiomyocyte nuclei are diploid up to 7 years of age (though higher levels of ploidy are observed following childhood cardiac disease) (Brodsky *et al*. 1994). Glucocorticoids play a vital role in the normal maturational changes that occur in cardiomyocytes before birth. The extent to which endogenous glucocorticoids directly affect cardiac maturation in the neonate remains unclear. Even more unclear is how ACT affects these maturational processes in heart structure and function, both before and after birth.

## Glucocorticoid action in the fetal heart

Glucocorticoids bind to intracellular receptors: the glucocorticoid receptor (GR or type II receptor) and the high-affinity mineralocorticoid receptor (MR or type I receptor). In mineralocorticoid target tissues, MR is co-expressed with 11β-HSD2, conferring mineralocorticoid specificity upon MR (Chapman *et al*. 2013). However, in certain MR-expressing tissues in which 11β-HSD2 is not co-expressed, most notably the heart and the hippocampus, MR is activated by cortisol/corticosterone. Moreover, because of its high affinity, cardiac MR is likely to be occupied even at the glucocorticoid nadir. Importantly, while betamethasone and dexamethasone (the synthetic glucocorticoids commonly used in ACT) potently activate GR, they are mineralocorticoid sparing, showing little activation of MR (we return to this below). Ligand-bound GR and MR translocate to the nucleus where they are active as transcription factors, triggering cascades of gene expression (Coutinho & Chapman 2011).

Glucocorticoids, through GR, promote structural, functional and metabolic remodelling in mouse fetal cardiomyocytes (Rog-Zielinska *et al*. 2013, 2015). Mice with global GR knockout have small and immature hearts that function poorly. The E/A wave ratio, a marker of cardiac maturity, is reduced in GR knockout mice and early systolic and diastolic function are impaired (Rog-Zielinska *et al*. 2013), similar to the preterm heart (reviewed in Rog-Zielinska *et al*. 2014). This functional immaturity goes hand-in-hand with structural immaturity (short and disorganised myofibrils, a lack of cardiomyocyte alignment in the outer ventricle wall) and biochemical immaturity (calcium handling, energy metabolism). Endogenous glucocorticoids directly impact cardiomyocytes. SMGRKO mice, with GR deficiency restricted to cardiomyocytes and vascular smooth muscle cells (VSMCs), replicate much of the cardiac phenotype of global GR knockout mice (Rog-Zielinska *et al*. 2013). SMGRKO mice have immature systolic function in late gestation, associated with short and disorganised myofibril structure (Rog-Zielinska *et al*. 2013). This is underpinned by reduced expression of genes encoding structural proteins myosin heavy chain (MHCα) and proteins vital for calcium handling (SERCA2, RYR2) and energy metabolism in late gestation (Rog-Zielinska *et al*. 2013).

Not all the actions of endogenous glucocorticoids upon the fetal heart are direct. The use of a cardiomyocyte-specific approach avoids the perturbation of blood pressure and suppression of the hypothalamic–pituitary–thyroid (HPA) axis associated with exogenous glucocorticoid administration. While SMGRKO mice reproduce much of the cardiac phenotype of global GR-knockout mice, some aspects differ. In contrast to global GR knockout mice, SMGRKO mice have normal-sized hearts, a normal E/A-wave ratio and normal cardiac expression of *Nppa* (encoding atrial natriuretic peptide, ANP) (Rog-Zielinska *et al*. 2013). This suggests that these deficits in the global knockout mice are attributable to lack of GR elsewhere than in cardiomyocytes and VSMC. Hemodynamic forces may account for at least some of these differences. Fetal ANP responds to volume stimuli and regulates blood pressure (Cameron & Ellmers 2003). The normal cardiac expression of ANP in SMGRKO fetuses suggests blood pressure is normal (as it is in adult SMGRKO mice (Richardson *et al*. 2017)). Glucocorticoids exert powerful pressor effects, including in the fetus (Unno *et al*. 1999, Forhead & Fowden 2004). Indeed, adrenal insufficiency is likely to be a factor in the haemodynamic instability that is common in preterm infants and which contributes to morbidity and mortality (Ng *et al*. 2004, Ibrahim *et al*. 2011). The non-cardiac sites for these important hemodynamic effects are currently unclear, though the fetal and placental vasculature are candidates. Antenatal dexamethasone treatment in rats dramatically reduces the extent and complexity of the vasculature within the labyrinth zone (the site of feto–maternal exchange), causing intrauterine growth restriction (IUGR) (Hewitt *et al*. 2006). In pregnant mice, corticosterone or dexamethasone treatment (at doses sufficient to cause IUGR) reduce placental vascularisation if administered in mid-gestation (E11–16) but not if administered from E14–E19, the time when glucocorticoid concentrations naturally rise in mice (Vaughan *et al*. 2012, 2013). Excessive glucocorticoid concentrations may also impact feto–placental vasculature in humans. In pregnant women, repeat courses of ACT starting at gestational ages ranging from 27 to 32 weeks impaired the normal gestational increase in villous capillarisation (Elfayomy & Almasry 2014). Similarly, glucocorticoid usage in pregnant women with asthma was associated with reduced fetal capillary length and volume (Mayhew *et al*. 2008). Given the crucial role of the placental vasculature in cardiac development (Thornburg *et al*. 2010), glucocorticoids very likely contribute to fetal cardiac maturation via effects on feto–placental vasculature. Support for this idea comes from a recent study in *Hsd11b2* *^−^* *^/^* *^−^* mice, a model of antenatal GC excess and IUGR. *Hsd11b2* *^−^* *^/^* *^−^* mice show abnormal cardiac maturation, with impaired heart function in late gestation (Wyrwoll *et al*. 2016). This is associated with restricted growth of fetal vessels in the placenta and reduced umbilical vein blood velocity (Wyrwoll *et al*. 2009, 2016). Restoration of the normal placental vasculature and blood flow restored normal heart function in *Hsd11b2* *^−^* *^/^* *^−^* mice (Wyrwoll *et al*. 2016).

The mechanisms by which glucocorticoids mature fetal cardiomyocytes have been investigated *in vitro.* Treatment of mouse fetal cardiomyocytes with either dexamethasone or corticosterone for 24 h promotes their structural, functional and biochemical maturation (Rog-Zielinska *et al*. 2015). GR targets in these cells include a number of transcription factors (Rog-Zielinska *et al*. 2015), supporting a cascade effect of GR activation. *In vivo* and *in vitro* dexamethasone rapidly induces PGC-1α, a critical regulator of cardiac mitochondrial capacity and vital for the functional and metabolic maturation of the fetal heart (Lai *et al*. 2008). Knockdown of PGC-1α in fetal cardiomyocytes abolished the glucocorticoid effects on mitochondrial O_2_ consumption and myofibril structure, suggesting it is a key mediator of glucocorticoid-induced maturation of cardiomyocyte structure and mitochondrial capacity (Rog-Zielinska *et al*. 2015). Whether PGC-1α mediates other aspects of glucocorticoid-induced cardiomyocyte maturation is unknown. However, PGC-1α knockdown in human embryonic stem cell-derived cardiomyocytes decreased mitochondrial content and activity (as expected) and also reduced concentrations of reactive oxygen species (ROS) and heightened vulnerability to metabolic stress (Birket *et al*. 2013). This suggests that glucocorticoids, via PGC-1α might increase ROS production in maturing cardiomyocytes. Other primary targets of GR in fetal cardiomyocytes include *Ppara*, *Klf15* and *Lipin1* (Rog-Zielinska *et al*. 2015). This points to a direct role for GR in promoting the capacity of cardiomyocytes for fatty acid oxidation as the preferred substrate for cardiac mitochondrial ATP generation, consistent with the increase in cardiac ATP concentration and ATP delivery to myofibrils induced by dexamethasone administration in late gestation rats (Tsuzuki *et al*. 2009, Mizuno *et al*. 2010). Other rapidly induced targets of GR in primary fetal mouse cardiomyocytes include *Dio2*, encoding deiodinase (D2) the enzyme that converts thyroxine (T_4_) into the more biologically active thyroid hormone, T_3_ (Rog-Zielinska *et al*. 2015). Thus, amplification of intracellular thyroid hormone action is part of the cascade of events initiated by GR activation and is consistent with evidence that at least some of the effects of the prepartum increase in glucocorticoids are mediated via T_3_ (Forhead & Fowden 2014). Indeed, cortisol increases plasma availability of T_3_ in the fetus by inducing hepatic expression of D1 (responsible for producing most of the circulating T_3_), and downregulates placental clearance of T_3_ by the D3 enzyme (Forhead & Fowden 2014, Jonker & Louey 2016). Many of the actions of glucocorticoid and thyroid hormones during fetal and neonatal heart maturation overlap. Both accelerate the switch from MHC-β to MHC-α and both increase ANP (van Tuyl *et al*. 2004, Chattergoon *et al*. 2012*a*) (these are indirect effects in the case of glucocorticoids (Rog-Zielinska *et al*. 2015)). *In vivo*, haemodynamic forces are changed by both glucocorticoids and thyroid hormones. Like synthetic glucocorticoids (Rog-Zielinska *et al*. 2014), *in vitro* and possibly *in vivo*, T_3_ is anti-proliferative in cardiomyocytes, promoting the switch to hypertrophic growth and increasing the population of terminally differentiated binucleated myocytes (Chattergoon *et al*. 2007, 2012*a*,*b*). If GR is precociously activated before the HPA axis has started to produce circulating fetal thyroid hormones in any substantial amount, this could limit maturation of fetal organs by glucocorticoids. This may be more important in rodents (where fetal thyroid hormone synthesis only initiates in late gestation) than in humans or in sheep models, where thyroid hormone synthesis occurs by mid-gestation (Forhead & Fowden 2014). Interestingly, while dexamethasone alone is ineffective in human induced pluripotent cell (iPSC)-derived cardiomyocytes, it acts in concert with T_3_ to improve the electrophysiology, bioenergetics and contractile force generation of the cells (Birket *et al*. 2015). Both T_3_ and dexamethasone are also required for T-tubule development in human iPSC-derived cardiomyocytes (Parikh *et al*. 2017). This inter-dependent relationship of thyroid hormone and glucocorticoids in cardiac maturation is an important consideration for ACT, where only glucocorticoid is administered.

## The transition to neonatal life: a role for glucocorticoids in cardiac adaption?

The transition to extra-uterine life is accompanied by loss of the placenta, a low-resistance vascular bed. In term birth, the associated circulatory changes rapidly increase peripheral resistance in the systemic circulation, with greater cardiac afterload and systemic arterial pressure. Blood and tissue oxygenation rapidly increase (partial pressure of oxygen in arterial blood increases over two-fold), as do plasma glucose and free fatty acid concentrations, to fuel the increased energy needs (Jonker & Louey 2016). Endogenous glucocorticoids are likely to be critical in facilitating these adaptions, although apart from metabolic effects, their role has been little explored. Interestingly, expression profiling and *in silico* transcription factor analysis in sheep heart across the perinatal period suggests glucocorticoids transiently suppress immune responses at birth and that they support metabolic changes during the transition from fetal to neonatal life (Richards *et al*. 2015).

In mice, cell-cycle arrest in cardiomyocytes occurs shortly after birth and is triggered by increased mitochondrial ROS production and oxidative DNA damage following birth (Puente *et al*. 2014). Transient hyperoxia in neonatal mice exacerbates oxidative DNA damage and accelerates cell-cycle arrest, whereas hypoxia has the opposite effect. Only hypoxia alters (increases) the heart-to-body weight ratio (Puente *et al*. 2014). Reduced cell number with hyperoxia is compensated by cardiomyocyte hypertrophy. Transient hyperoxia in neonatal rats causes left ventricular hypertrophy in adulthood and increases vulnerability to pressure overload, with severe heart failure in those exposed to transient high O_2_ at birth (Bertagnolli *et al*. 2014). The mechanism was not explored, but reduced cardiomyocyte endowment as a result of premature cell-cycle exit, is associated with pathological cardiac hypertrophy in adult rats (Porrello *et al*. 2009). Glucocorticoids may promote this process: in neonatal rats, administration of dexamethasone from P1 to P3 decreased cardiomyocyte proliferation and increased binucleation at P4, with a reduction in cardiomyocyte number and an increase in heart/body weight ratio (presumably as a result of cardiomyocyte hypertrophy) apparent by 2 weeks of age (Gay *et al*. 2015). Dexamethasone induces oxidative stress in neonatal rat hearts (Adler *et al*. 2010). Whether this is mediated through PGC-1α will be interesting to establish, given the role of PGC-1α in mitochondrial ROS production in human embryonic stem cell-derived cardiomyocytes (Birket *et al*. 2013). Deletion of GR in cardiomyocytes/VSMC increases cardiomyocyte proliferation in neonatal mice and increases heart weight, without associated cardiomyocyte hypertrophy (Richardson *et al*. 2017). This is consistent with GR activation normally restraining cardiomyocyte number in neonatal mice. Glucocorticoid activation of cardiac MR may have the opposite effect. At subpressor doses, cortisol stimulates cardiomyocyte proliferation in fetal sheep without affecting binucleation (Giraud *et al*. 2006). This hyperplastic effect can be blocked by intra-pericardial antagonism of MR (Feng *et al*. 2013). Similarly, spironalactone antagonism of MR from birth reduced adult heart weight in mice, independently of cardiomyocyte GR (Richardson *et al*. 2017). This is consistent with endogenous glucocorticoid action via MR being pro-proliferative in perinatal cardiomyocytes.

## The heart in preterm infants

Preterm birth occurs before the organs have had sufficient time to accrue mass, and when they are structurally and functionally immature. Thus, in preterm infants, heart mass (relative to body weight) is reduced at birth (Aye *et al*. 2017). Moreover, if driven by cardiac load, oxygenation and arterial pressure, then terminal differentiation of cardiomyocytes will likely occur in the same time scale in the preterm heart as in the term. In an important recent study, hearts from preterm infants who died 1–42 days following birth were compared with appropriately grown stillborn infants (20–40 weeks of gestation). Birth *per se* reduced markers of cardiomyocyte proliferation compared to age-matched stillborns (Bensley *et al*. 2018). Therefore, preterm birth prompts the cessation of cardiomyocyte proliferation, plausibly as a result of the oxygen-induced DNA damage discussed above (Puente *et al*. 2014). This potentially reduces cardiomyocyte endowment in preterm infants compared to the term-born infants, resulting in maladaptive structural remodelling in the neonatal period. Alterations in cardiac geometry and mechanics are already apparent in the preterm-born infant and child (Lee *et al*. 1992, Aye *et al*. 2017). The reduced heart mass after preterm birth becomes normalised by disproportionate cardiac hypertrophy and increased left ventricular mass in subsequent ‘catch-up’ growth in the early postnatal period (Kozak-Barany *et al*. 2001, Aye *et al*. 2017), with differences in cardiac structure and function persisting in adulthood (reviewed in Bensley *et al*. 2016, Le *et al*. 2018). Preterm-born adults show increased left ventricular mass (independent of blood pressure), thickened ventricular walls and displaced apex (Lewandowski *et al*. 2013). Studies in preterm lambs demonstrate that catch-up growth occurs by cardiomyocyte hypertrophy: cardiomyocyte volume is increased despite no differences in absolute or relative heart weight (Bensley *et al*. 2010). Similarly, in hypoplastic mouse hearts, myocardial mass is normalised by accelerated cardiomyocyte hypertrophy (Drenckhahn *et al*. 2015). Thus, myocyte hypertrophy can compensate for fewer cardiomyocytes to maintain mass. However, in the very and extremely preterm infant, the reduction in cardiomyocyte number may be too great for this compensatory mechanism to normalise heart size: left ventricular mass is reduced in adults born extremely preterm (Kowalski *et al*. 2016) and in children born very preterm (Mohlkert *et al*. 2018).

Ontogenetically determined cardiomyocyte number (or endowment) is an important factor in vulnerability to cardiac disease (Levkau *et al*. 2008). In a large study of over 2.6 million individuals born in Sweden between 1987 and 2012, gestational age prior to 32 weeks at delivery was strongly associated with risk of heart failure in childhood and early adulthood. The risk was increased 17-fold in those born at <28 weeks and 4-fold if born at 28–31 weeks of gestation (Carr *et al*. 2017). This suggests a vulnerability to heart failure with preterm birth, with a reduced threshold at which an insult will trigger heart failure. Reduced cardiomyocyte endowment with compensatory hypertrophy may explain this vulnerability, with subsequent greater stress on individual myocytes. Stress and/or ageing is associated with polyploidy in cardiomyocyte nuclei (Anatskaya *et al*. 2009, Senyo *et al*. 2013). Polyploidy may be a mechanism to allow larger cardiomyocytes to fulfil metabolic and functional demands (Schoenfelder & Fox 2015). In humans, polyploidy is likely to arise in childhood and is strongly associated with impaired cardiac function and pathological hypertrophy (Brodsky *et al*. 1991, 1994). Whether preterm hearts show higher levels of ploidy is unknown. However, myocyte ploidy is increased in preterm sheep (Bensley *et al*. 2010) consistent with polyploidy as an indicator of greater susceptibility to cardiac stress/load in the preterm-born adult. Fibrosis, seen in adults born preterm, may occur secondarily to cardiomyocyte loss.

## ACT: what are we doing to the heart?

Although preterm birth is strongly associated with increased risk of cardiovascular disease in later life, the evidence to date suggests that effects of preterm birth on cardiac morphology and function are independent of antenatal corticosteroid exposure (Dalziel *et al*. 2005, Aye *et al*. 2017). While both are predicted to have similar effects (e.g. reduced cardiomyocyte endowment, cardiomyocyte hypertrophy), ACT is likely to be a minor contributor compared to preterm birth itself. Indeed, should preterm birth occur, in the short term, ACT may enhance cardiomyocyte function directly through rapid effects on metabolic and structural maturation and possibly indirectly by improving haemodynamic stability. Glucocorticoids increase haemodynamic load in the fetus as well as the neonate (Unno *et al*. 1999), likely to affect cardiomyocyte proliferation and/or maturation independently of direct effects of glucocorticoids in cardiomyocytes. Indeed, systemic haemodynamic instability (hypotension and low systemic blood flow) is inversely related to gestational age at birth (du Plessis 2009) and is a cause of mortality in some preterm infants (Ng *et al*. 2004, Kluckow 2005, Sehgal 2011, Rog-Zielinska *et al*. 2014). In fetuses >29 weeks gestational age, heart rate was rapidly (within 24 h) and transiently decreased following betamethasone (Mulder *et al*. 2004). This was ascribed to the baroreceptor reflex in which an increase in blood pressure very rapidly triggers a decrease in heart rate, causing blood pressure to fall. This did not occur in younger fetuses (Mulder *et al*. 2004). Thus, ACT has gestational age-specific effects on the developing fetus, consistent with the idea of developmental windows of susceptibility to the maturational effects of glucocorticoids, including in the heart, dependent on the stage of organ development at the time of glucocorticoid exposure: organogenesis, growth or maturation. This warrants further research.

Of concern, around half of women exposed to a single course of ACT do not deliver their babies within the optimal 2- to 7-day time window (Kemp *et al*. 2016). If delivered more than 7 days after a single course of ACT, the risk of perinatal death is increased two-fold and neonatal death three-fold (McLaughlin *et al*. 2003). The reasons may be multi-factorial, but studies in sheep support the idea that heart function is compromised by excessive glucocorticoid exposure *in utero*. A modest but chronic maternal hypercortisolaemia in late gestation sheep altered the trajectory of myocyte maturation and increased expression of calcium signalling genes (Richards *et al*. 2014), consistent with a short-term benefit. However, continuation of maternal hypercortisolaemia to term resulted in a high incidence of stillbirth (Keller-Wood *et al*. 2014). This was associated with reduced cardiac mitochondrial number and function and increased cardiac expression of hypoxia-response genes, though not the hypoxia master regulators, HIF1α or ARNT themselves (Richards *et al*. 2014). Chronic maternal hypercortisolaemia also altered the fetal ECG and depressed aortic pressure and heart rate immediately prior to delivery (Antolic *et al*. 2018). The authors speculate that glucocorticoid-induced alterations in fetal cardiac metabolism and/or ion homeostasis contribute to cardiac dysfunction, creating vulnerability to death during labour and/or delivery. In mice, a number of ion channels are direct targets of GR in fetal cardiomyocytes (Rog-Zielinska *et al*. 2015) and transgenic over-expression of GR in cardiomyocytes of adult mice causes ion-channel remodelling (Sainte-Marie *et al*. 2007). MR over-expression in cardiomyocytes also causes ion-channel remodelling (Ouvrard-Pascaud *et al*. 2005). Teasing apart the relative contributions of GR and MR (and indeed, the balance between MR and GR (Richardson *et al*. 2016)) to the complex effects of endogenous and exogenous glucocorticoids in the heart will be important in understanding how excessive glucocorticoid exposure *in utero* increases risk of perinatal death. Further research in this area is needed.

There are longer-term concerns. The association between excessive exposure to glucocorticoid *in utero* (in term birth) and cardiovascular disease in adulthood is well known (Seckl & Holmes 2007). Indeed, excessive antenatal glucocorticoid exposure is considered as a key factor in the fetal origins of disease hypothesis (Seckl & Holmes 2007, Fowden & Forhead 2015, Fowden *et al*. 2016). It is, as yet, unclear if the short-term exposure to high concentrations of potent glucocorticoids used in ACT adversely affects the heart if birth does not occur within 7 days. We and others have reviewed a body of (sometimes conflicting) evidence from sheep and rodents suggesting glucocorticoid treatment reduces cardiomyocyte proliferation and promotes cardiomyocyte hypertrophy in the fetal and/or neonatal perinatal heart (Porrello *et al*. 2008, Rog-Zielinska *et al*. 2014, Richardson *et al*. 2016), beneficial in the short-term in promoting heart function and survival, but possibly at the expense of adult cardiovascular resilience. As discussed earlier, cardiomyocyte endowment is a key factor in susceptibility to cardiac disease particularly in heart failure where cardiomyocyte loss contributes to development and progression of the disease (Rayment *et al*. 1999). People born at term who are exposed to excess glucocorticoids *in utero* may start life with fewer cardiomyocytes, contributing to risk of cardiovascular disease. More information on this vital question is required, in particular whether and how endogenous and exogenous glucocorticoids increase or reduce cardiomyocyte number at birth.

What are the effects of ACT upon endogenous glucocorticoid production and how might this affect the fetal and/or neonatal heart? Maternal stress, perinatal adversity and exposure to inappropriately timed or excessive concentrations of glucocorticoids prenatally are all associated with life-long alterations in HPA axis activity (Seckl & Holmes 2007, Waffarn & Davis 2012, Moisiadis & Matthews 2014). Relative adrenal insufficiency may occur in preterm neonates (especially if delivered prior to the initiation of *de novo* adrenocortical glucocorticoid synthesis at 28 weeks gestational age) or could result from ACT. The adrenal gland undergoes extensive remodelling immediately after term birth (Keene 1927) with regression of the fetal zone and maturation of the zona fasciculata. How this is affected in preterm infants and by ACT is unclear. However, what is clear is that both the maternal and the fetal HPA axis are suppressed following ACT (reviewed in Waffarn & Davis 2012). Corticosteroids administered in ACT persist in the fetal circulation for up to 3 days following the last dose (Ballard *et al*. 1975, Kajantie *et al*. 2004, Nykanen *et al*. 2007). However, cortisol concentration in the neonate remains depressed well after clearance of administered steroid from the circulation: a meta-analysis of 49 studies concluded that both basal cortisol and stress-induced cortisol release are suppressed in the preterm infant (Tegethoff *et al*. 2009). Furthermore, and while basal cortisol concentration recovers within 2 weeks of delivery, the cortisol response to pain is suppressed for at least 4 weeks and possibly considerably longer (Tegethoff *et al*. 2009). In sheep, three courses of betamethasone reduced late gestation fetal cortisol concentration, which remained suppressed 6 weeks postnatally (Li *et al*. 2013). Pituitary expression of *POMC* (encoding the precursor to ACTH) was reduced up to 12 weeks postnatally (Li *et al*. 2013). ACT also affects stress reactivity and HPA axis regulation in those infants who are delivered at or near term. In one study, newborns exposed to ACT but delivered near or at term were unable to mount a cortisol stress response, in contrast to non-exposed newborns (Schaffer *et al*. 2009). On the other hand, another study reported an increase in stress reactivity of cortisol in term-born neonates after ACT exposure (Davis *et al*. 2011). The reasons for the differences are unclear, but may relate to the conditions of testing or gestational age at ACT exposure. In an animal model, the ability of neonatal rats to mount a glucocorticoid response to stress depended on developmental age and was stressor specific (Walker 1991). HPA axis abnormalities persist: school-age children exposed to ACT but born at term had abnormal diurnal cortisol release: they lacked the normal cortisol awakening response and had a flattened diurnal rhythm in salivary cortisol concentration compared with non-exposed term-born children (Edelmann *et al*. 2016).

These effects of ACT upon endogenous glucocorticoid production will have two consequences in the fetal and neonatal heart: First, in the short-term the HPA axis suppression will deprive cardiac MR of their glucocorticoid ligand. This will severely alter the cardiac MR/GR balance in favour of GR, for example, favouring the anti-proliferative effect of GR activation over the pro-proliferative effects of MR activation (Richardson *et al*. 2016). A similar consideration applies to MR in the hippocampus: here, the severe adverse psychological/psychotic effects of dexamethasone in children treated for acute lymphoblastic leukaemia can be ameliorated by co-administration of cortisol, for replacement at the MR (Warris *et al*. 2016). Secondly, persistent suppression of the fetal HPA axis following ACT may also deprive GR as well as MR of stage-appropriate levels of its endogenous ligand. ACT, even in the term born, may therefore alter the trajectory of fetal and neonatal heart maturation through changes in endogenous glucocorticoid production.

## Concluding remarks

ACT is an established and effective therapy to improve lung function in preterm infants, to reduce neonatal morbidity and mortality. However, increasing evidence suggests it is not always harm-free, particularly in infants delivered at or near term (Althabe *et al*. 2015). The jury is still out on whether ACT alters the risk of late-onset cardiac disease, but given the complex effects of corticosteroids on haemodynamics and fetal and neonatal heart maturation, it would be surprising if it does not impact on the trajectory of perinatal heart maturation, subsequent cardiac growth and vulnerability to insult.

More knowledge is urgently needed to inform future refinements to ACT. Current treatment protocols were largely established decades ago and are based on limited knowledge regarding optimal formulation, timing of dosage and efficacy at different gestational ages. A ‘one size fits all’ dosage applies, irrespective of maternal weight or gestational age. It seems likely that different processes during cardiac development may be affected by ACT at different gestational ages. With the increasing use of ACT in preterm infants as young as 22 weeks, it is important to elucidate how glucocorticoids may impact heart structure and function at different times. The manner of cardiomyocyte proliferation shapes the heart. If ACT alters cardiomyocyte proliferation, particularly in a region-dependent manner, then both shape and function will be affected (Foglia & Poss 2016). There may be sex differences in cardiac susceptibility to ACT. Certainly evidence from sheep supports a sex difference in lung maturation, with a lower dose of betamethasone required for survival of female lambs compared to male (De Matteo *et al*. 2010). Mice with GR knockout in cardiomyocytes show sex differences in cardiomyocyte hypertrophy (Richardson *et al*. 2017) and in susceptibility to cardiac disease (Oakley *et al*. 2013). Answers to these questions should help inform and refine future use of ACT.

## Declaration of interest

The authors declare that there is no conflict of interest that could be perceived as prejudicing the impartiality of this review.

## Funding

This work did not receive any specific grant from any funding agency in the public, commercial or not-for-profit sector.
